# Effect of *Bifidobacterium longum* on cognition and microbiota in post-stroke patients: a single-blinded, controlled trial

**DOI:** 10.7150/ijms.124024

**Published:** 2026-02-11

**Authors:** Shu-Ting Lin, Te-Hsuan Tung, Yen-Nung Lin, Feng-Hang Chang, Yu Zhi Lian, Chien-Hung Lai, Tsan-Hon Liou, Chi-Chang Huang, Ya-Ling Chen, Jane C.-J. Chao

**Affiliations:** 1 School of Nutrition and Health Sciences, College of Nutrition, Taipei Medical University, Taipei, Taiwan.; 2 Pittsburgh Institute for Neurodegenerative Diseases, University of Pittsburgh, Pittsburgh, PA, USA.; 3 Department of Physical Medicine and Rehabilitation, Wan Fang Hospital, Taipei Medical University, Taipei, Taiwan.; 4 Graduate Institute of Injury Prevention and Control, College of Public Health, Taipei Medical University, Taipei, Taiwan.; 5 Department of Physical Medicine and Rehabilitation, Taipei Medical University Hospital, Taipei, Taiwan.; 6 Department of Physical Medicine and Rehabilitation, Shuang Ho Hospital, Taipei Medical University, New Taipei, Taiwan.; 7 Graduate Institute of Metabolism and Obesity Sciences, College of Nutrition, Taipei Medical University, Taipei, Taiwan.; 8 Graduate Institute of Sports Science, National Taiwan Sport University, Taoyuan, Taiwan.; 9 Master Program in Global Health and Health Security, College of Public Health, Taipei Medical University, Taipei, Taiwan.; 10 Nutrition Research Center, Taipei Medical University Hospital, Taipei, Taiwan.; 11 TMU Research Center for Digestive Medicine, Taipei Medical University, Taipei, Taiwan.

**Keywords:** *Bifidobacterium longum* OLP-01, stroke, cognitive function, physical performance, gut microbiota

## Abstract

Stroke is the major leading cause of death globally, and has a high disability rate. Malnutrition, cognitive dysfunction, motor impairment, and gut dysbiosis are observed in stroke patients. Our study explored the effects of *Bifidobacterium longum* OLP-01, a probiotic from an Olympics gold medalist, on cognitive function and gut microbiota as the primary outcomes, and on nutritional status and physical performance as the secondary outcomes in post-stroke patients. The study was a single-blinded, randomized, placebo-controlled trial. We recruited 22 post-stroke patients. Participants received either placebo (placebo group, *n* = 10) or 2 × 10^10^ colony-forming units (CFU) of *Bifidobacterium longum* OLP-01 powder (OLP-01 group, *n* = 12) daily for 12 weeks. Nutritional status was assessed using Mini Nutritional Assessment (MNA). Cognitive function was evaluated by Montreal Cognitive Assessment (MoCA), Trail-Making Tests, and Stroop Color Naming Test. Physical performance was measured using Timed Up and Go Test and 6-Minute Walk Test. Blood and stool samples were collected for biochemical and gut microbiota analyses. The OLP-01 group increased MoCA scores, decreased time to completion of Trail-Making Tests, and had greater accuracy on incongruent in Stroop Color Naming Test, but decreased MNA scores within the normal ranges. The cognitive-related gut microbiota strain showed differences between the two groups. Supplementation of *Bifidobacterium longum* OLP-01 at a dose of 2 × 10^10^ CFU daily for 12 weeks improved cognitive function and changes gut microbiota, but did not alter physical performance in post-stroke patients.

## Introduction

Stroke is a condition where the central nervous system is damaged due to vascular injury, and leads to acute and focal neurological deficit 1. The World Health Organization reported that stroke was the second leading cause of death worldwide, and caused 6.09 million deaths in 2020 2. Thrombosis and hematoma further lead to brain injury and neurological deficits such as cognitive and motor impairment 3. Other consequences of stroke including dysphagia, surgery, and physical or emotional stress increased the risk of malnutrition in patients 4-7.

The gut-brain axis refers to the bidirectional communication between the central nervous system and the gastrointestinal tract 8. Gut dysbiosis accompanied by leaky gut could disrupt the integrity of the blood-brain barrier, and lead to elevated lipopolysaccharides. Increased lipopolysaccharides could activate the nuclear factor-κB signaling pathway, and further induce inflammatory responses and destroy blood-brain barrier integrity, which might influence brain function 9, 10. Furthermore, inflammatory responses could also cause skeletal muscle atrophy via the gut-muscle axis 11. Previous studies showed that stroke patients were particularly vulnerable to gut dysbiosis which could worsen cognitive impairment 12.

Probiotic supplementation such as *Bifidobacterium longum* has shown promise in regulating gut microbiota and improving cognitive function and digestion in humans 13, 14. Probiotic *Bifidobacterium longum* is gram-positive prokaryotes, and naturally colonizes human gastrointestinal tract 15. The healthy elderly with resistance exercise and supplementation of *Bifidobacterium spp.* (1.25 × 10^10^ colony-forming units (CFU)/day) for 12 weeks improved cognitive function 16. The previous *in vitro* study showed that *Bifidobacterium longum* could be beneficial to cognitive function via the gut-brain axis model, and the results revealed that *Bifidobacterium longum* at a dose of 10 mg/mL could regulate mitochondrial membrane potential and further ameliorate cell damage of human neuroblastoma SHSY-5Y cells caused by oxidative stress in the co-cultured model of human colorectal adenocarcinoma Caco-2 and SHSY-5Y cells 17. Another animal study also demonstrated that supplementation with *Bifidobacterium longum* at a dose of 1 × 10^9^ CFU/day for 1 month alleviated aging-dependent cognitive impairment in 18 months-old 5XFAD-transgenic mice by changing fecal microbiota composition, suppressing pro-inflammatory cytokines in the colon and hippocampus, and increasing brain-derived neurotrophic factor (BDNF) expression in the hippocampus 18. Similar findings about the improvement of oral administration with *Bifidobacterium longum* (1-2 × 10^9^ CFU/day) for 5 days to 12 weeks on declined cognitive function were reported in mice via the modification of gut microbiota, the inhibition of brain inflammation, and the up-regulation of BDNF 19-21, providing evidence for the promising effects on the improvement of cognitive impairment by the supplementation of *Bifidobacterium longum*.

Although supplementation with *Bifidobacterium longum* OLP-01 at the equivalent dose of 1-1.5 × 10^10^ CFU/day for humans improved exercise performance in mice after 4 weeks 22 and runners after 5 weeks 23, and increased the abundance of gut probiotics in runners, however the effects of *Bifidobacterium longum* in post-stroke patients are not well understood. The aims of this study were to investigate the effects of *Bifidobacterium longum* OLP-01 on cognitive function and gut microbiota as the primary outcomes, and on nutritional status and physical performance as the secondary outcomes in post-stroke patients.

## Materials and Methods

### Trial design

The clinical trial (NCT05477732, last updated: July 28, 2022), a single-blinded (participants), randomized, controlled trial, was conducted between 2021 and 2022. The 1st participant was recruited on March 24th in 2021, and the last participant was recruited on January 26th in 2022. Post-stroke patients were recruited for all the following criteria: (1) stroke diagnosed for more than 3 months, (2) age between 20 and 75 years, and (3) stable rehabilitation in outpatient clinics. Patients were excluded for any of the following criteria: (1) severe disabilities (e.g., aphasia, dementia, or depression), (2) BMI ≥ 35 kg/m^2^, (3) cancer therapy in the past three months, (4) other severe disease to interfere with their participation in this study, or (5) emotional or mental condition to be unable to cooperate with the examination and treatment. Participants were recruited from the Department of Physical Medicine and Rehabilitation at 3 Taipei Medical University affiliated hospitals: Taipei Medical University Hospital, Wan Fang Hospital, and Shuang Ho Hospital. The trial was followed the Consolidated Standards of Reporting Trials. The study protocol and informed consent were approved by the Taipei Medical University Joint Institutional Review Board (N202011038), and informed consent was obtained from all participants.

**Figure 1:**
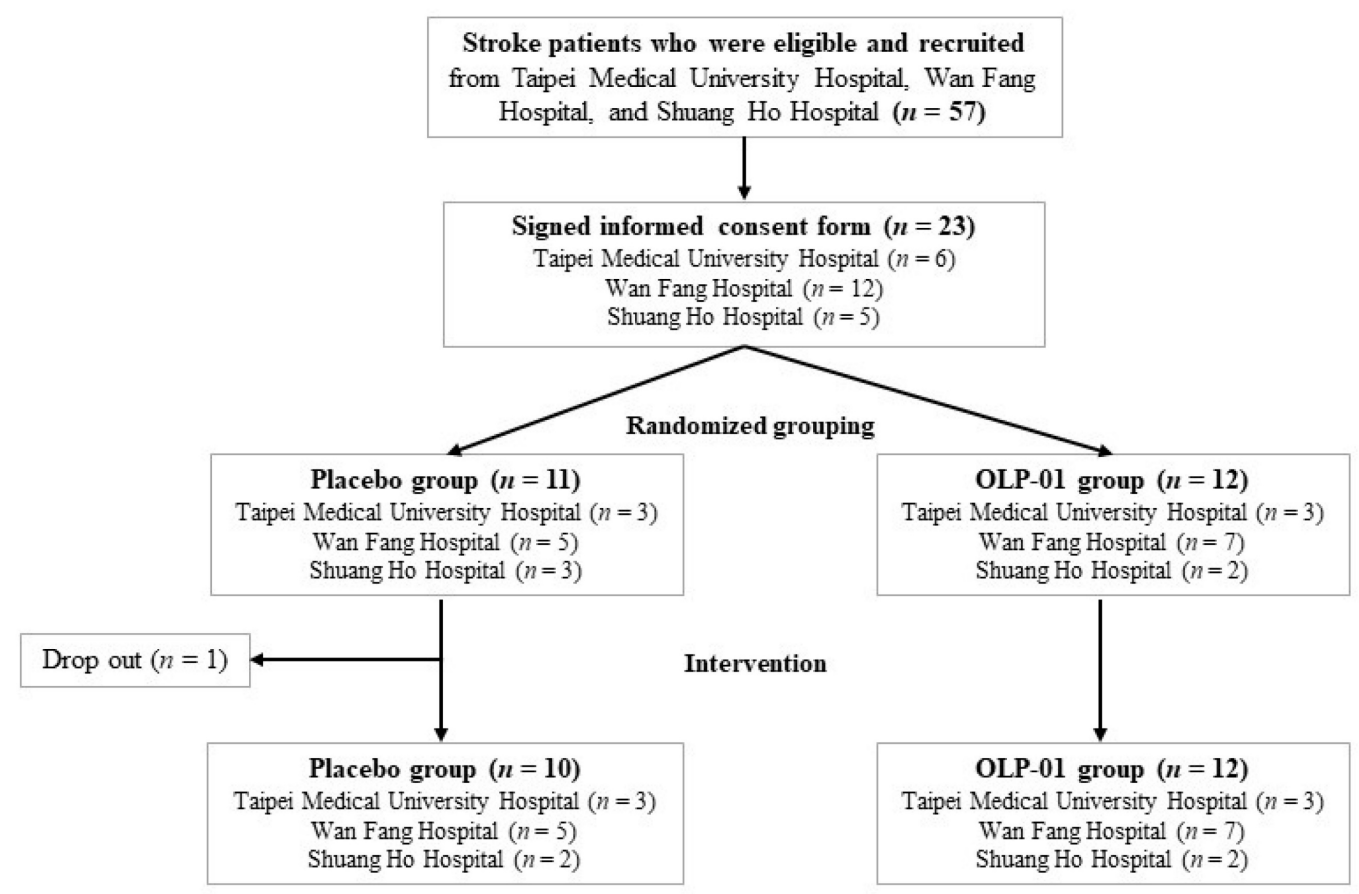
Flowchart of subject recruitment from 3 hospitals.

The sample size of 12 participants in each group was determined based on the effect size (d = 0.95), α error (α = 0.05), and 73% power. The flowchart of subject recruitment from 3 hospitals is shown in Figure 1. Participants were randomly allocated to the placebo (*n* = 10) or OLP-01 group (*n* = 12), and received rehabilitation during 12-week intervention period. Participants in the placebo group were given one pack (2 g/pack) of the powder mixture with isomalto-oligosaccharides, maltodextrin, and corn starch (FacLac Biotech, Hsinchu, Taiwan), whereas the OLP-01 group were given one pack (1 × 10^10^ CFU/2 g/pack) of *Bifidobacterium longum* OLP-01 powder (FacLac Biotech, Hsinchu, Taiwan) purified from a Taiwanese weightlifting gold medalist 23, both groups taken a total 2 packs per day before breakfast and dinner. The dose of *Bifidobacterium longum* OLP-01 powder in one pack was referred to the effective dosage for improving exercise performance in the previous studies 22, 23. We increased the dosage and the supplementation period in the previous healthy younger human study 23 because considering the conditions of our subjects (older and post-stroke patients). Participants were asked to maintain their medication, dietary habits, lifestyle, and rehabilitation exercise, and avoid consuming any supplement such as probiotics (except for the assigned probiotics) or prebiotics that might affect gut microbiota at least one month before and during the entire intervention period. Frequency of daily intervention intake, medication and rehabilitation exercise including types and frequency weekly were recorded. Compliance rate was calculated by the following formula: compliance rate=actual frequency intake/ assigned frequency of intake×100%. The compliance rate for all subjects, placebo group, or OLP-01 group was 94.9%, 96.5%, or 93.4%, respectively. No adverse effects were reported during the intervention period.

### Clinical, anthropometric, biochemical, and nutritional assessments

Stroke related medical information including period after stroke diagnosed, type of stroke, side of lesion, and use of aid were recorded from the medical record. Medical history, medication, and lifestyle information were collected by the questionnaire. At weeks 0 and 12, blood pressure and pulse were assessed by an electronic sphygmomanometer. Anthropometric data such as body mass index (BMI), circumferences, and waist-to-hip ratio were measured. Muscle mass, body fat, and basal metabolic rate (BMR) were evaluated using Inbody 120 (Upwards Biosystems, Taipei, Taiwan). The type of stroke was defined as follows: (1) ischemic stroke was an episode of neurological dysfunction caused by focal cerebral, spinal, or retinal infarction 24, (2) hemorrhagic stroke was a brain injury attributable to acute blood extravasation into the brain parenchyma from a ruptured cerebral blood vessel 25, and (3) subarachnoid hemorrhage was identified as the nontraumatic abrupt onset of severe headache or altered level of consciousness associated with blood in the subarachnoid space 26.

Blood biochemical variables including protein status, lipid profile, glycemic profile, complete blood counts, white blood cell differential counts, and C-reactive protein (CRP) were analyzed using an automated analyzer (Cobas8000 e602 module, Roche Holding AG, Basel, Switzerland). Inflammatory cytokines such as tumor necrosis factor-α (TNF-α) (430204, BioLegend, San Diego, CA, USA), interleukin-6 (IL-6) (DY206-05, R&D Systems, Inc., Minneapolis, MN, USA), and IL-10 (430607, BioLegend, San Diego, CA, USA) were measured using commercial kits.

Nutritional status was assessed using Mini Nutritional Assessment (MNA) 27. Dietary intake was collected by a well-trained investigator with nutrition background, and evaluated by 3-day 24-hour dietary record including 2 weekdays and 1 weekend at weeks 0 and 12. Dietary intake for energy and nutrients was then calculated by food composition analysis using the Cofit software (Cofit Healthcare Inc., Taipei, Taiwan), which is a database application for the intake of energy and nutrients with food composition information collected from the databases of local foods, food manufacturers, and restaurants 28, 29.

### Evaluation of cognitive function and physical performance

Cognitive function was assessed using Montreal Cognitive Assessment (MoCA), Trail-Making Tests A and B (TMT A and B), and Stroop Color Naming Test. The MoCA evaluates visuospatial ability, executive function, naming, memory, attention, language abstraction, delayed recall, and orientation, and was established as a reliable and valid tool for detecting cognitive impairment in individuals with stroke, cardiovascular risk factors, or subarachnoid hemorrhage 30. The TMT A and B measured visual search, processing speed, cognitive flexibility, and executive function, and the TMT A consists of 24 numbered circles and the TMT B contains numbers and words that should be connected or switched in a specified order within a set time limit 31. The Stroop Color Naming Test assesses cognitive interference, attention, processing speed, cognitive flexibility, and working memory 32, including congruent and incongruent parts, subjects should read colored words printed in the same or different colors with the ink in which they are written 32. The time to complete the tasks, number of correct responses, and correct percentage were recorded.

Physical performance was measured using Timed Up and Go Test (TUGT) and 6-Minute Walk Test. The TUGT evaluates balance, walking ability, and the risk of falls 30, subjects were asked to rise from a chair, walk 3 meters, turn around, return to the chair, and sit down, and the time of completion was recorded 33, 34. The 6-Minute Walk Test assesses walking endurance 35, subjects were requested to walk on a 10-meter course for 6 minutes, and the walking distance was recorded 35.

### Analysis of gut microbiota

Stool samples were collected using a commercial kit (BIOTOOLS Co., Ltd., Taipei, Taiwan), and analyzed by Core Laboratory of Human Microbiome, Taipei Medical University. The QIAamp Fast DNA Stool Mini Kit (Qiagen Benelux B.V., Venlo, Netherlands) was used, and the library preparation was followed the protocol of 16S rRNA gene amplicons for the Illumina MiSeq System. All raw amplicon sequencing reads and associated metadata have been deposited in the National Center for Biotechnology Information Sequence Read Archive repository under BioProject PRJNA1514980. Gene-specific sequences targeted the 16S V3 and V4 regions, and the demultiplexed paired reads were removed using cutadapt (v1.12). The filtered reads were processed using the DADA2 R package (v1.14.1) in R software (v3.6.1), and followed by the workflow described in previous study 36.

### Statistical analysis

Values were presented as mean (standard deviation, SD) or numbers (%). Categorical data were analyzed using chi-squared test, and continuous data were compared using paired *t* test for the comparison within the group or Student's *t* test for the comparison between the groups using SPSS 18.0 software (SPSS Inc., Chicago, IL, USA). The taxonomy of gut microbiota was conducted using the SILVA database (v138). Multiple sequence alignment of the structural variations was performed using DECIPHER (v2.14.0), and a phylogenetic tree was constructed from the alignment using phangorn (v2.5.5). The resulting phylogenetic tree was consolidated into a phyloseq object, and community analyses were performed using phyloseq (v1.30.0). Alpha-diversity indices were calculated using the estimate_richness function from the phyloseq package. Kruskal-Wallis or Wilcoxon test was performed to compare between the groups. Beta-diversity was analyzed. Unique fraction (UniFrac) distances were calculated using the GUniFrac package (v1.1). Principal coordinate analysis (PCoA) ordination on UniFrac distances was performed, and the adonis and betadisper functions from the vegan package (v2.5.6) were used for the dissimilarity of composition between the groups and the homogeneity of dispersion, respectively. Enrichment analysis between the groups was evaluated using the linear discriminant analysis (LDA) effect size (LEfSe), and logarithmic LDA score greater than 2 was significant. The results were shown as a cladogram using GraPhlAn. The *p*-value ≤ 0.05 was significant.

## Results

### Baseline characteristics

The mean age of all participants was 57.0 (SD = 9.8) years, and there were 12 (54.5%) men and 10 (45.5%) women (Table 1). The age range of our recruited participants was from 33 to 70 years old (data not shown), but the gender, age, BMI, chronic disease, lifestyle, and MoCA score were similar between the groups, and shown no significant differences (Table 1). The period after stroke diagnosed among the participants was ranged from 6 to 120 months (data not shown), and the period, type of stroke, side of lesions, and used of aid were not statistically different between the groups (Table 2).

### Anthropometric, clinical, biochemical, and nutritional status

Anthropometric data (Table 3), blood pressure, blood protein status, lipid and glycemic profiles, CRP, IL-6, and IL-10 (Table 4) were not different within and between the groups at week 0 and 12. The levels of TNF-α in the placebo group were decreased after 12 weeks (Table 4). Complete blood counts were not different in the same group at week 0 and 12 (Table 5). However, neutrophils were lower in the OLP-01 group than those in the placebo group at week 12, and lymphocytes in the OLP-01 group were higher than those in the placebo group at weeks 0 and 12. Other blood cell counts and the change of blood cell counts between week 12 and 0 were not different between both groups.

At week 12, MNA total score in the OLP-01 group was lower compared to the placebo group (*p* = 0.025) (Table 4). However, MNA assessment score in the OLP-01 group was decreased compared to that in the placebo group (*p* = 0.016), and the difference in the change of MNA assessment score between the groups was also significant (*p* = 0.047). The mean of MNA total and assessment scores in both groups was remained in normal nutritional status.

Intakes of protein and fat (g) in the OLP-01 group was higher, but carbohydrate intake (% energy) was lower at week 0 compared the placebo group (Table 6). Subjects in the placebo group increased fat intake at week 12 compared to week 1. Neither significant differences in the OLP-01 group at different time points nor significant changes for intakes of energy, macronutrients, and dietary fiber were found between two groups. Neither significant differences in the same group at different time points nor significant changes for micronutrient intakes were observed (Table 7).

### Improved cognitive function by *B. longum* OLP-01

After 12-week supplementation with *B. longum* OLP-01, cognitive function tests showed improvement in the OLP-01 group (Table 8). Specifically, MoCA total score and scores in language and delayed recall sections were increased. Additionally, the time for completing TMT A and TMT B tests was reduced, and the correct ratio of incongruent in the Stroop Color Naming Test was increased.

### No beneficial effects of *B. longum* OLP-01 on physical performance

Physical performance of TUGT and 6-Minute Walk Test showed no differences either in the placebo or the OLP-01 group between week 0 and week 12 (Table 9). However, the changes for the distance of 6-Minute Walk Test after 12 weeks were different (*p* = 0.024) between the placebo (positive changes) and the OLP-01 group (negative changes).

### Altered abundance of gut microbiota by *B. longum* OLP-01

**Figure 2:**
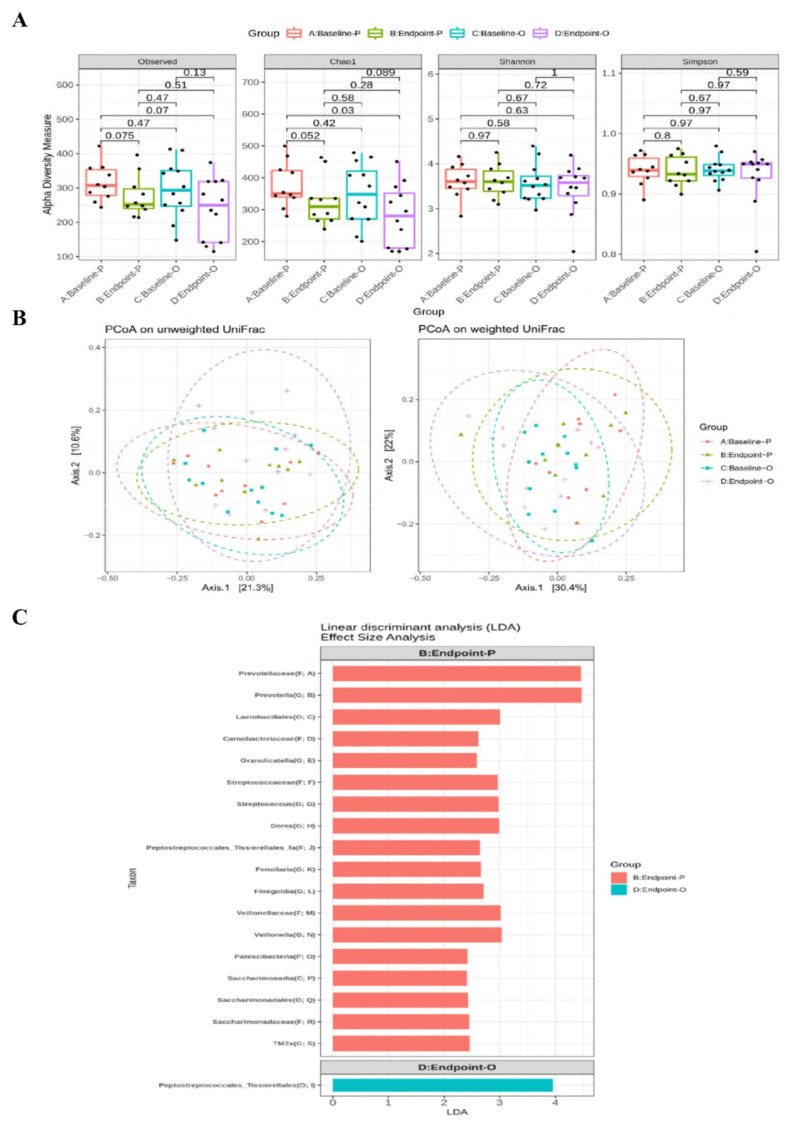
Diversity and LEfSe analysis of gut microbiota between the placebo and the OLP-01 groups. **A**, The analysis of α-diversity; **B**, the analysis of β-diversity; **C**, enrichment analysis using LEfSe method. The first letter in the parenthesis of the taxon indicates the abbreviation of the taxonomic ranks. A: baseline of the placebo group (*n* = 10), B: endpoint of the placebo group (*n* = 10), C: baseline of the OLP-01 group (*n* = 12), D: endpoint of the OLP-01 group (*n* = 12). C, class; F, family; G, genus; LEfSe, linear discriminant analysis effect size; O, order; OUT, operational taxonomic unit; P, phylum; PCoA, principal coordinate analysis; UniFrac, unique fraction.

The relative abundance of main phylum (Supplementary Figure 1), family (Supplementary Figure 2), and genus (Supplementary Figure 3) of gut microbiota were not altered at week 12 between the two groups. The α-diversity of Chao1 index tended to be decreased (*p* = 0.052) at week 12 in the placebo group compared to that at week 0 (Figure 2A). There were no differences in β-diversity between or within the two groups after 12 weeks (Figure 2B). The LEfSe analysis revealed that certain bacterial order (*Lactobacillales*), families (*Prevotellaceae*, *Streptococcaceae*, and *Veillonellaceae*), and genera (*Prevotella*, *Streptococcus*, *Dorea*, and *Veillonella*) were more abundant in the placebo group, whereas the Order *Peptostreptococcales_Tissierellales* was more abundant in the OLP-01 group at week 12 (Figure 2C).

## Discussion

The prevalence of malnutrition in stroke patients ranged widely from 6.1% to 62% attributed by different nutrition assessment method, time of assessment, and characteristics of the subjects such as dysphagia 37. Our study showed that 10% and 25% post-stroke patients were at risk of malnutrition at week 12 in the placebo and the OLP-01 group, respectively, MNA scores was even higher in the placebo group after 12 weeks, suggesting such significant difference could be due to the placebo effect.

The improvement of cognitive function is a critical concern for post-stroke patients, probiotic was considered strongly potential as supplementation with *Bifidobacteria*-containing probiotics for at least 4 weeks improved cognitive function in murine models 38, 39. Several studies have assessed the association between probiotics supplement and cognitive function, but the effects varied due to different confounders including age, physical status, and strain, dosage, and supplementation duration of probiotics 10, 23. Neurotrophic factors such as BDNF and nerve growth factor were vital for neurogenesis, neuron survival, synaptic plasticity, and associated with learning and memory function 10 and link between gut microbiota and brain function was observed due to BDNF as a crucial role in this gut-brain axis 40, as microbial composition affected intra-cerebral neurotrophic factors and neurotransmitters, indicating the potential for probiotics to improve cognitive function 41. Probiotic supplementation (*B. longum, B. breve,* and* B. infantis*) at the dose of ≥ 1 × 10^9^ CFU/kg/day each strain for 4 weeks increased spine density, dendritic arborization, and the extent of long-term potentiation in the hippocampus along with elevated hippocampal BDNF in rats 38. A randomized, double-blind, placebo-controlled trial found that 12-week *Bifidobacterium* supplementation (*B. bifidum* BGN4 and *B. longum* BORI) at a total dose of 1 × 10^9^ CFU/day improved cognitive function and mental stress along with elevated serum BDNF in healthy elderly 10. Our study revealed that *B. longum* OLP-01 supplementation increased MoCA total score, reduced completion time in TMT A and TMT B test, and increased correct ratios in the incongruent Stroop Color Naming Test. These results supported that visual skills, task processing ability, and memory function were improved by *B. longum* OLP-01 supplementation, such mechanism should be further determined.

Supplementation of *B. longum* OLP-01 at the dose of ≥ 2.05 × 10^9^ CFU/kg/day for 4 weeks showed anti-fatigue properties with reduced serum lactate and improved grip strength and endurance in mice after acute exercise 22, and combining 5-week *B. longum* OLP-01 supplementation with 3-week regular exercise training improved endurance exercise performance for middle- and long-distance runners 23. However, such effects were not demonstrated in balance progress (TUGT) and endurance (6-Minute Walk Test) after probiotics supplementation, probably due to higher ratios of walking aid use and long duration since stroke diagnosis in the OLP-01 group, and the absence of exercise practice in this study. The data suggested that the beneficial effects of *B. longum* OLP-01 supplementation may be due to synergistic effects with regular exercise.

Probiotics and/or their metabolites are thought to be the primary contributors in their beneficial effects on gut microbiota and physiological functions. Supplementation with *Bifidobacterium* strains for 6 weeks increased the abundance of *Ruminococcaceae* UCG-009 and *Ruminococcaceae* UCG-010 in mice 42. Additionally, fecal transplant gavage from young mice rich in probiotics and short-chain fatty acids (SCFA) significantly improved behavioral recovery in aged post-stroke mice by enhancing the integrity of gut barrier and inhibiting the inflammatory response in the gut and brain, which was associated with enriched *Bifidobacteriaceae* and *Clostridiaceae* 43. These studies suggested that the composition of gut microbiota can be changed by probiotics supplementation. However, few alterations in the gut microbiota were observed after 12-week *B. longum* OLP-01 supplementation in our study. We speculated that stroke may affect gut microbiota composition, as previous animal study was treated with probiotics in the early stage of stroke, whereas our study recruited patients who had been diagnosed with stroke for a longer period 43. Additionally, the previous study used antibiotics to disrupt the original gut microbiota before probiotics treatment, which may be difficult to be conducted in clinical trials 43.

Our study showed that the OLP-01 group had lower abundance of *Prevotellaceae* and *Prevotella* at week 12 compared to the placebo group. A previous study found that healthy elderly receiving 12-week probiotics supplementation (*B. bifidum* BGN4 and *B. longum* BORI) had lower levels of *Eubacterium* and *Prevotellaceae*, which were pro-inflammatory microbiota associated with autoimmune disease and chronic intestinal inflammation 11, 44, 45. The abundance of *Prevotella* was inversely correlated with cognitive function and MoCA scores in stroke patients, in the domains of delayed recall, orientation, and abstraction 46. The data suggested that less *Prevotellaceae* and *Prevotella* could be correlated with better cognitive function.

This is first clinical trial to investigate the effects of *B. longum* OLP-01 supplementation in post-stroke patients, but there were several limitations. It was a single-blinded trial with a small sample size, which may increase the bias risk. Furthermore, this study was performed during the COVID-19 pandemic, and we faced challenges for the access to hospitals and reduced patients' willingness to participate in this study, increasing the difficulty for subject recruitment. The impacts of gender, specific stroke type, or hemorrhagic site were not further investigated in this study because of the limited number of the subjects. Additionally, few subjects were unable to stand stably or walk for long distances, which may affect the accuracy of physical performance tests. The diet was not controlled during the experimental period, and the differences in dietary intake could be a confounder for the composition of gut microbiota, which may influence the results of this study. We did not record and further assessed the precise location and extent of brain lesion among the participants, which could also influence the results. The molecular mechanism of probiotics supplementation on cognitive function remains unclear, and SCFA concentration played a role in improving inflammatory status was not assessed 44.

## Conclusion

Supplementation with *B. longum* OLP-01 improves cognitive function, specifically memory function, and decreases the abundance of *Prevotellaceae* and *Prevotella* in post-stroke patients.

## Supplementary Material

Supplementary figures.

## Figures and Tables

**Table 1:** Baseline characteristics of post-stroke patients

Characteristics	All subjects (*n* = 22)	Placebo (*n* = 10)	OLP-01 (*n* = 12)	*p-*value
Male/Female, *n* (%)	12 (54.5)/10 (45.5)	6 (60.0)/4 (40.0)	6 (50.0)/6 (50.0)	0.639
Age, years (SD)	57.0 (9.8)	60.2 (8.2)	54.4 (10.5)	0.172
Height, cm (SD)	164.1 (9.4)	163.3 (7.7)	164.8 (10.9)	0.726
Weight, kg (SD)	68.0 (14.0)	67.4 (10.3)	68.5 (17.0)	0.860
BMI, kg/m^2^ (SD)	25.0 (3.3)	25.3 (3.7)	24.9 (3.0)	0.780
Diabetes, *n* (%)				
Yes	3 (13.6)	0 (0.0)	3 (25.0)	0.089
No	9 (86.4)	10 (100.0)	9 (75.0)	
Hypertension, *n* (%)				
Yes	12 (54.5)	6 (60.0)	6 (50.0)	0.639
No	10 (45.5)	4 (40.0)	6 (50.0)	
Dyslipidemia, *n* (%)				
Yes	4 (18.2)	2 (20.0)	2 (16.7)	0.840
No	18 (81.8)	8 (80.0)	10 (83.3)	
Cigarette smoking, *n* (%)				
Yes	2 (9.1)	2 (20.0)	0 (0.0)	0.267
No	20 (90.9)	8 (80.0)	12 (100.0)	
Alcohol drinking, *n* (%)				
Yes	3 (13.6)	2 (20.0)	1 (8.3)	0.427
No	19 (86.4)	8 (80.0)	11 (91.7)	
Exercise frequency,* n* (%)				
None	3 (13.6)	1 (10.0)	2 (16.7)	0.716
< 150 min/week	10 (45.5)	4 (40.0)	6 (50.0)	
≥ 150 min/week	9 (40.9)	5 (50.0)	4 (33.3)	
MoCA, total score (SD)	23.8 (4.9)	25.2 (5.0)	22.7 (4.6)	0.232

Values are mean (SD) or* n* (%). BMI: body mass index, MoCA: Montreal Cognitive Assessment.

**Table 2:** Stroke related characteristics of post-stroke patients at the baseine

Baseline characteristics	All subjects (*n* = 22)	Placebo (*n* = 10)	OLP-01 (*n* = 12)	*p-*value
Period after stroke diagnosed, month (SD)	38.3 (33.9)	25.6 (21.8)	48.8 (39.3)	0.112
Type of stroke, *n* (%)				
Ischemic stroke	10 (45.4)	6 (60.0)	4 (33.3)	0.421
Hemorrhagic stroke	8 (36.4)	3 (30.0)	5 (41.7)	
Subarachnoid hemorrhage	4 (18.2)	1 (10.0)	3 (25.0)	
Side of lesion, *n* (%)				
Left	15 (68.2)	6 (60.0)	9 (75.0)	0.452
Right	7 (31.8)	4 (40.0)	3 (25.0)	
Use of aid, *n* (%)				
Yes	15 (68.2)	5 (50.0)	10 (83.3)	0.095
No	7 (31.8)	5 (50.0)	2 (16.7)	

Values are mean (SD) or *n* (%).

**Table 3:** Anthropometric characteristics and basal metabolic rate of post-stroke patients

Characteristics	Placebo (*n* = 10)			OLP-01 (*n* = 11-12)			*p-*value*
	Week 0	Week 12	Change	Week 0	Week 12	Change	
Weight, kg	67.4 (10.3)	68.2 (10.6)	0.8 (2.6)	68.5 (17.0)	68.5 (17.2)	0.1 (2.9)	0.533
BMI, kg/m^2^	25.3 (3.7)	25.6 (4.0)	0.4 (1.0)	24.9 (3.0)	24.8 (3.3)	-0.1 (1.1)	0.377
Waist circumference, cm	91.3 (7.2)	92.2 (9.4)	0.9 (5.6)	92.9 (11.4)	93.3 (11.5)	0.4 (7.1)	0.873
Hip circumference, cm	101.2 (7.6)	99.6 (7.2)	-1.6 (2.9)	100.3 (5.6)	100.3 (6.9)	0.0 (3.3)	0.245
Waist/hip ratio	0.90 (0.07)	0.90 (0.06)	0.00 (0.05)	0.90 (0.05)	0.90 (0.05)	0.00 (0.03)	0.759
Mid-upper arm circumference, cm	29.3 (3.4)	28.6 (3.3)	-0.7 (2.5)	29.4 (4.2)	29.2 (3.6)	-0.2 (3.8)	0.739
Calf circumference, cm	37.0 (3.9)	36.9 (4.1)	-0.1 (1.4)	36.4 (4.2)	36.0 (4.4)	-0.4 (1.4)	0.636
Muscle mass, kg	25.8 (4.4)	26.1 (4.6)	0.2 (0.6)	26.1 (8.9)	26.8 (8.9)	0.8 (1.7)	0.339
Body fat, %	29.7 (6.7)	30.2 (7.9)	0.4 (2.6)	31.9 (6.9)	30.0 (5.9)	-1.9 (5.0)	0.207
BMR, kcal	1393 (166)	1402 (178)	9 (47)	1362 (317)	1388 (324)	26 (48)	0.411

Values are mean (SD). *The* p-*values represent the changes between the two groups. No significant differences (*p* > 0.05) were found in the same group at different time points and between the two groups at weeks 0 and 12. BMI: body mass index, BMR: basal metabolic rate.

**Table 4:** Blood pressure, blood biochemical variables, and nutritional assessment of post-stroke patients

Variables	Placebo (*n* = 10)			OLP-01 (*n* = 11-12)			*p-*value^*^
	Week 0	Week 12	Change	Week 0	Week 12	Change	
Blood pressure and blood biochemical variables							
Systolic pressure, mmHg (SD)	123.9 (9.5)	123.8 (10.7)	-0.1 (7.2)	117.0 (6.5)	120.0 (8.5)	3.0 (9.4)	0.404
Diastolic pressure, mmHg (SD)	77.0 (12.8)	79.8 (13.6)	2.8 (5.1)	76.9 (8.7)	78.9 (8.0)	2.0 (8.4)	0.794
Pulse, bpm (SD)	73.8 (11.9)	70.6 (9.1)	-3.2 (6.5)	75.5 (11.9)	76.5 (10.1)	1.0 (8.8)	0.225
Albumin, g/L (SD)	46.1 (3.6)	46.4 (1.8)	0.3 (2.6)	46.4 (3.7)	45.8 (2.0)	-0.5 (2.9)	0.492
Prealbumin, g/L (SD)	0.3 (0.1)	0.3 (0.0)	0.0 (0.0)	0.3 (0.1)	0.3 (0.1)	0.0 (0.0)	0.399
Transferrin, g/L (SD)	2.4 (0.4)	2.4 (0.4)	0.0 (0.2)	2.6 (0.8)	2.6 (0.6)	0.0 (0.2)	0.626
TG, mmol/L (SD)	13.5 (4.5)	15.6 (8.3)	2.2 (7.8)	14.5 (6.1)	16.8 (9.7)	2.3 (7.6)	0.965
Total cholesterol, mmol/L (SD)	4.56 (0.68)	4.63 (0.76)	0.06 (0.54)	5.05 (1.02)	4.99 (0.91)	-0.06 (0.39)	0.536
HDL-C, mmol/L (SD)	1.27 (0.22)	1.26 (0.34)	-0.01 (0.21)	1.26 (0.23)	1.23 (0.21)	-0.02 (0.13)	0.835
LDL-C, mmol/L (SD)	2.87 (0.59)	2.93 (0.60)	0.06 (0.45)	3.21 (0.91)	3.22 (0.88)	0.01 (0.29)	0.785
Fasting glucose, mmol/L (SD)	5.0 (0.4)	5.1 (0.8)	0.1 (0.9)	5.0 (0.6)	5.0 (0.5)	0.1 (0.4)	0.814
HbA_1c_, % (SD)	5.6 (0.3)	5.6 (0.4)	0.0 (0.3)	5.6 (0.4)	5.6 (0.4)	0.0 (0.2)	0.769
CRP, mg/L (SD)	1.34 (0.72)	2.00 (2.17)	0.66 (2.30)	2.17 (2.55)	2.31 (2.50)	0.14 (1.09)	0.507
TNF-α, pg/mL (SD)	8.5 (6.7)	4.0 (7.2)^†^	-4.5 (6.1)	6.3 (8.0)	4.0 (4.4)	-2.3 (7.1)	0.458
IL-6, pg/mL (SD)	15.5 (29.9)	17.6 (19.3)	2.1 (28.5)	4.5 (7.2)	17.0 (22.3)	12.5 (22.1)	0.359
IL-10, pg/mL (SD)	1.5 (1.1)	2.1 (1.6)	0.6 (1.6)	1.0 (0.9)	1.3 (0.8)	0.3 (0.9)	0.618
Mini Nutritional Assessment (MNA)							
MNA total score (SD)	25.5 (2.0)	26.3 (1.6)	0.8 (1.3)	25.2 (2.2)	24.5 (1.8)^‡^	-0.7 (2.2)	0.066
Normal status, *n* (%)	7 (70.0)	9 (90.0)	2	8 (66.7)	9 (75.0)	1	N/A
At risk of malnutrition,* n* (%)	3 (30.0)	1 (10.0)	-2	4 (33.3)	3 (25.0)	-1	N/A
MNA screening score (SD)	12.8 (1.0)	13.0 (1.1)	0.2 (0.8)	12.6±0.9	12.5 (1.4)	-0.1 (1.5)	0.547
MNA assessment score (SD)	12.7 (1.3)	13.3 (0.9)^†^	0.6 (0.8)	12.6±1.6	12.0 (1.3)^‡^	-0.6 (1.5)	0.047

Values are mean (SD) or n (%). ^*^The P-values represent the changes between the two groups. †Represents significant differences (*p* < 0.05) in the same group at different time points. ‡Represents differences (*p* < 0.05) between the two groups at week 12. TG: triglycerides, HDL-C: high-density lipoprotein cholesterol, LDL-C: low-density lipoprotein cholesterol, HbA_1c_: glycated hemoglobin, CRP: C-reactive protein, TNF-α: tumor necrosis factor-α, IL-6: interleukin-6, IL-10: interleukin-10, N/A: not applicable.

**Table 5:** Complete blood counts of post-stroke patients

Complete blood counts	Placebo (*n* = 10)			OLP-01 (*n* = 11)			*p-*value*
	Week 0	Week 12	Change	Week 0	Week 12	Change	
WBC, 10^9^/L	6.0 (1.9)	6.3 (2.2)	0.4 (0.8)	6.0 (0.9)	6.0 (0.7)	0.0 (0.9)	0.346
RBC, 10^12^/L	4.7 (0.5)	4.7 (0.5)	0.0 (0.2)	4.6 (0.5)	4.7 (0.4)	0.0 (0.2)	0.568
HGB, g/L	145.7 (13.1)	144.0 (13.5)	-1.7 (6.2)	138.6 (12.5)	140.0 (13.4)	1.4 (5.1)	0.227
HCT, %	42.6 (3.6)	42.4 (3.9)	-0.3 (1.9)	41.2 (3.4)	41.6 (3.6)	0.3 (1.6)	0.428
PLT, 10^9^/L	194 (91)	197 (101)	4 (26)	250 (81)	243 (76)	-7 (25)	0.350
MCV, fL	91.0 (3.9)	91.1 (3.7)	0.1 (1.3)	88.9 (4.8)	89.3 (4.6)	0.4 (1.7)	0.659
MCH, fmol	1.9 (0.1)	1.9 (0.1)	0.0 (0.0)	1.9 (0.1)	1.9 (0.1)	0.0 (0.1)	0.638
MCHC, g/L	341.3 (5.7)	340.0 (7.5)	-1.3 (6.0)	336.8 (9.9)	336.2 (7.4)	-0.6 (7.1)	0.820
NEUT, number fraction	0.62 (0.06)	0.63 (0.08)	0.01 (0.07)	0.53 (0.12)	0.52 (0.10)^‡^	-0.01 (0.06)	0.536
LYM, number fraction	0.27 (0.06)	0.26 (0.05)	-0.01 (0.06)	0.36 (0.11)^†^	0.36 (0.10)^‡^	0.00 (0.05)	0.791
MONO, number fraction	0.08 (0.01)	0.07 (0.02)	-0.01 (0.02)	0.07 (0.01)	0.07 (0.01)	0.00 (0.01)	0.070
EOS, number fraction	0.03 (0.02)	0.03 (0.03)	0.00 (0.03)	0.03 (0.02)	0.03 (0.02)	0.00 (0.01)	0.900
BASO, number fraction	0.01 (0.00)	0.01 (0.00)	0.00 (0.00)	0.01 (0.00)	0.01 (0.00)	0.00 (0.00)	0.591

Values are mean (SD). *The *p-*values represent the changes between the two groups. No significant differences (*p* > 0.05) were found in the same group at different time points. ^†^Represents significant differences (*p* < 0.05) between the two groups at week 0.^ ‡^Represents significant differences (*p* < 0.05) between the two groups at week 12. WBC: white blood cells, RBC: red blood cells, HGB: hemoglobin, HCT: hematocrit, PLT: platelets, MCV: mean corpuscular volume, MCH: mean corpuscular hemoglobin, MCHC: mean corpuscular hemoglobin concentration, NEUT: neutrophils, LYM: lymphocytes, MONO: monocytes, EOS: eosinophils, BASO: basophils.

**Table 6:** Dietary intakes of energy, macronutrients, and dietary fiber in post-stroke patients

Dietary intake	Placebo (*n* = 10)			OLP-01 (*n* = 12)			*p-*value^*^
	Week 0	Week 12	Change	Week 0	Week 12	Change	
Energy, kcal	1514 (317)	1612 (261)	98 (233)	1683 (375)	1668 (397)	-15 (335)	0.379
Protein							
Protein, g	63.2 (14.7)	68.1 (15.0)	4.9 (13.3)	77.6 (16.1)^‡^	76.0 (20.4)	-1.6 (17.3)	0.347
Protein, % energy	16.8 (2.5)	17.0 (3.1)	0.1 (2.6)	18.6 (2.6)	18.1 (1.7)	-0.5 (2.6)	0.570
Fat							
Fat, g	53.8 (9.7)	65.0 (16.3)^†^	11.2 (15.5)	72.1 (22.8)^‡^	70.5 (20.1)	-1.6 (19.4)	0.108
Fat, % energy	32.4 (4.6)	36.0 (5.1)	3.6 (5.8)	38.0 (4.9)^‡^	37.8 (3.8)	-0.3 (4.1)	0.082
Carbohydrate (Carb)							
Carb, g	197.2 (56.5)	188.7 (36.9)	-8.5 (34.2)	184.3 (38.5)	186.0 (41.8)	1.7 (32.4)	0.483
Carb, % energy	51.5 (7.0)	47.1 (7.2)	-4.5 (8.2)	44.1 (4.6)^‡^	44.9 (3.8)	0.8 (4.2)	0.067
Dietary fiber, g	13 (5)	14 (7)	2 (6)	15 (6)	14 (4)	-1 (5)	0.366

Values are mean (SD). ^*^The *p-*values represent the changes between the two groups. †Represents significant differences (*p* < 0.05) in the same group at different time points. ‡Represents significant differences (*p* < 0.05) between the two groups at week 0. No significant differences (*p* > 0.05) were found between the two groups at week 12. Carb: carbohydrate.

**Table 7:** Dietary intakes of vitamins and minerals in post-stroke patients

Micronutrient	DRIs	Placebo (*n* = 10)			OLP-01 (*n* = 12)			*p-*value^*^
		Week 0	Week 12	Change	Week 0	Week 12	Change	
Vitamins								
Vitamin B_1_, mg	M: 1.2 F: 0.9	1.1 (0.7)	1.3 (0.6)	0.2 (0.6)	1.2 (0.5)	1.2 (0.6)	0.0 (0.4)	0.390
Vitamin B_2_, mg	M: 1.3 F: 1.0	1.2 (0.9)	1.1 (0.4)	-0.0 (0.5)	1.3 (0.7)	1.5 (0.9)	0.2 (0.5)	0.352
Vitamin B_6_, mg	M: 1.6 F: 1.6	1.4 (0.7)	1.4 (0.6)	0.0 (0.5)	1.8 (1.0)	1.8 (1.2)	-0.1 (0.5)	0.669
Vitamin B_12_, μg	2.4	2.6 (1.5)	3.2 (1.7)	0.6 (1.9)	4.5 (2.4)^†^	4.8 (2.6)	0.3 (1.8)	0.781
Folate, μg	400	235 (61)	304 (89)	69 (110)	326 (191)	326 (219)	1 (87)	0.117
Niacin, mg NE	M: 16 F: 14	11 (6)	14 (4)	3 (4)	15 (7)	15 (8)	0 (4)	0.193
Vitamin C, mg	100	110 (67)	104 (79)	-6 (80)	140 (54)	138 (73)	-2 (69)	0.898
Vitamin A, μg-RE	M: 600 F: 500	819 (604)	730 (386)	-89 (884)	1239 (627)	1168 (560)^‡^	-71 (583)	0.955
Vitamin E, mg-αTE	12	10 (7)	9 (3)	-1 (5)	19 (19)	19 (23)	1 (14)	0.760
Minerals								
Potassium, mg		1887 (729)	1995 (661)	108 (418)	2169 (672)	2274 (572)	105 (559)	0.988
Calcium, mg	1000	452 (226)	467 (201)	14 (242)	516 (19)	616 (264)	100 (189)	0.359
Magnesium, mg	M: 360 F: 310	202 (80)	219 (73)	17 (37)	234 (83)	245 (82)	11 (78)	0.807
Phosphate, mg	800	838 (272)	923 (282)	85 (185)	917 (242)	995 (321)	78 (243)	0.945
Iron, mg	10	8 (3)	9 (3)	2 (3)	13 (7)^†^	13 (7)	1 (4)	0.512
Zinc, mg	M: 15 F: 12	8 (2)	9 (2)	0 (2)	11 (5)	11 (6)	0 (3)	0.816

Values are mean (SD). ^*^The *p-*values represent the changes between the two groups. No significant differences (*p* > 0.05) were found in the same group at different time points. ^†^Represents significant differences (*p* < 0.05) between the two groups at week 0.^ ‡^Represents significant differences (*p* < 0.05) between the two groups at week 12. DRIs: Dietary Reference Intakes (Taiwan).

**Table 8:** Cognitive function tests of post-stroke patients

Cognitive tests	Placebo (*n* = 10)			OLP-01 (*n* = 12)			*p-*value^*^
	Week 0	Week 12	Change	Week 0	Week 12	Change	
MoCA total score	25.2 (5.0)	25.3 (5.1)	0.1 (1.9)	22.7 (4.6)	25.2 (3.7)^ †^	2.5 (2.1)	0.010
Visuospatial/executive score	4.2 (1.0)	4.7 (0.5)	0.5 (0.7)	4.2 (0.8)	4.7 (0.7)	0.5 (0.8)	1.000
Naming score	2.8 (0.6)	3.0 (0.0)	0.2 (0.6)	3.0 (0.0)	2.9 (0.3)	-0.1 (0.3)	0.179
Attention score	5.2 (1.0)	5.4 (1.0)	0.2 (1.0)	5.1 (1.2)	5.6 (0.9)	0.5 (0.9)	0.476
Language score	2.6 (0.5)	2.3 (0.8)	-0.3 (0.7)	2.1 (0.8)	2.7 (0.5)^ †^	0.6 (0.7)	0.006
Abstraction score	1.8 (0.6)	1.5 (0.7)	-0.3 (0.8)	1.8 (0.4)	1.8 (0.4)	0.0 (0.6)	0.336
Delayed recall score	2.9 (2.1)	3.2 (2.0)	0.3 (1.1)	1.5 (1.6)	2.5 (1.6)^ †^	1.0 (1.4)	0.212
Orientation score	5.7 (0.7)	5.2 (1.2)	-0.5 (0.7)	5.0 (1.5)	5.0 (1.5)	0.0 (0.7)	0.123
Trail Making Test							
A, second	67 (45)	62 (36)	-5 (16)	82 (52)	60 (37)^ †^	-22 (25)	0.083
B, second	165 (115)	148 (99)	-17 (68)	144 (76)	114 (53)^ †^	-30 (36)	0.566
Stroop Color Naming Test-congruent							
Total, *n*	61.2 (24.4)	72.8 (37.3)	11.6 (21.4)	65.5 (19.3)	69.8 (22.9)	4.3 (11.9)	0.326
Correct, *n*	61.1 (24.4)	72.4 (37.5)	11.3 (21.4)	65.3 (19.5)	69.8 (22.9)	4.5 (11.6)	0.354
Correct ratio, %	99.8 (0.5)	99.2 (1.2)	-0.6 (1.2)	99.6 (1.2)	100.0 (0.0)	0.4 (1.2)	0.079
Stroop Color Naming Test-incongruent							
Total, *n*	33.3 (12.6)	30.6 (15.0)	-2.7 (7.5)	36.0 (15.8)	36.8 (20.4)	0.8 (15.0)	0.516
Correct, *n*	31.8 (12.5)	29.4 (15.2)	-2.4 (8.1)	34.6 (17.0)	36.0 (21.1)	1.4 (15.0)	0.479
Correct ratio, %	94.7 (4.5)	94.9 (5.3)	0.2 (3.9)	92.8 (9.7)	95.7 (6.6)^ †^	2.9 (4.4)	0.143

Values are mean (SD). ^*^The *p-*values represent the changes between the two groups. ^†^Represents significant differences (*p* < 0.05) in the same group at different time points.

**Table 9:** Physical performance of post-stroke patients

Physical performance	Placebo (*n* = 10)			OLP-01 (*n* = 8-9)			*p-*value^*^
	Week 0	Week 12	Change	Week 0	Week 12	Change	
Timed Up and Go Test, second	28.1 (24.7)	28.9 (29.4)	0.8 (8.3)	42.2 (45.4)	35.7 (32.3)	-6.5 (14.4)	0.184
6-Minute Walk Test, meter	230 (156)	245 (155)	15 (20)	217 (157)	206 (142)	-11 (25)	0.024

Values are mean (SD). ^*^The *p-*values represent the changes between the two groups. No differences (*p* > 0.05) were found in the same group at different time points.

## Data Availability

The data in this study are available in this article and Additional files.

## Abbreviations

BMI: body mass index
BMR: basal metabolic rate
CFU: colony-forming units
CRP: C-reactive protein
IL: interleukin
LDA: linear discriminant analysis
LEfSe: linear discriminant analysis effect size
MNA: Mini Nutritional Assessment
MoCA: Montreal Cognitive Assessment
PCoA: principal coordinate analysis
TMT A and B: Trail-Making Tests A and B
TNF-α: tumor necrosis factor-α
TUGT: Timed Up and Go Test
UniFrac: unique fraction

## References

[1] Murphy SJ, Werring DJ (2020). Stroke: causes and clinical features. Medicine.

[2] GBD 2019 Diseases and Injuries Collaborators (2020). Global burden of 369 diseases and injuries in 204 countries and territories, 1990-2019: a systematic analysis for the Global Burden of Disease Study 2019. Lancet.

[3] Donkor ES (2018). Stroke in the 21st century: a snapshot of the burden, epidemiology, and quality of life. Stroke Res Treat.

[4] Al-Qazzaz NK, Ali SH, Ahmad SA, Islam S, Mohamad K (2014). Cognitive impairment and memory dysfunction after a stroke diagnosis: a post-stroke memory assessment. Neuropsychiatr Dis Treat.

[5] Langhorne P, Coupar F, Pollock A (2009). Motor recovery after stroke: a systematic review. Lancet Neurol.

[6] Alaverdashvili M, Caine S, Li X, Hackett MJ, Bradley MP, Nichol H (2018). Protein-energy malnutrition exacerbates stroke-induced forelimb abnormalities and dampens neuroinflammation. Transl Stroke Res.

[7] Koszewicz M, Jaroch J, Brzecka A, Ejma M, Budrewicz S, Mikhaleva L (2021). Dysbiosis is one of the risk factor for stroke and cognitive impairment and potential target for treatment. Pharmacol Res.

[8] Arya AK, Hu B (2018). Brain-gut axis after stroke. Brain Circ.

[9] Yang X, Yu D, Xue L, Li H, Du J (2020). Probiotics modulate the microbiota-gut-brain axis and improve memory deficits in aged SAMP8 mice. Acta Pharm Sin B.

[10] Kim C-S, Cha J, Sim M, Jung S, Chun WY, Baik HW (2021). Probiotic supplementation improves cognitive function and mood with changes in gut microbiota in community-dwelling older adults: a randomized, double-blind, placebo-controlled, multicenter trial. J Gerontol A Biol Sci Med Sci.

[11] Grosicki GJ, Fielding RA, Lustgarten MS (2018). Gut microbiota contribute to age-related changes in skeletal muscle size, composition, and function: biological basis for a gut-muscle axis. Calcif Tissue Int.

[12] Wen SW, Wong CH (2017). An unexplored brain-gut microbiota axis in stroke. Gut Microbes.

[13] Markowiak P, Śliżewska K (2017). Effects of probiotics, prebiotics, and synbiotics on human health. Nutrients.

[14] Wang J, Ji H (2019). Influence of probiotics on dietary protein digestion and utilization in the gastrointestinal tract. Curr Protein Pept Sci.

[15] Schell MA, Karmirantzou M, Snel B, Vilanova D, Berger B, Pessi G (2002). The genome sequence of *Bifidobacterium longum* reflects its adaptation to the human gastrointestinal tract. Proc Nat Acad Sci USA.

[16] Inoue T, Kobayashi Y, Mori N, Sakagawa M, Xiao JZ, Moritani T (2018). Effect of combined bifidobacteria supplementation and resistance training on cognitive function, body composition and bowel habits of healthy elderly subjects. Benef Microbes.

[17] Ferrari S, Galla R, Mulè S, Rosso G, Brovero A, Macchi V (2023). The Role of *Bifidobacterium bifidum* novaBBF7, *Bifidobacterium longum* novaBLG2 and *Lactobacillus paracasei* TJB8 to improve mechanisms linked to neuronal cells protection against oxidative condition in a gut-brain axis model. Int J Mol Sci.

[18] Lee HJ, Lee KE, Kim JK, Kim DH (2019). Suppression of gut dysbiosis by *Bifidobacterium longum* alleviates cognitive decline in 5XFAD transgenic and aged mice. Sci Rep.

[19] Ni Y, Yang X, Zheng L, Wang Z, Wu L, Jiang J (2019). *Lactobacillus* and *Bifidobacterium* improves physiological function and cognitive ability in aged mice by the regulation of gut microbiota. Mol Nutr Food Res.

[20] Lee DY, Shin YJ, Kim JK, Jang HM, Joo MK, Kim DH (2021). Alleviation of cognitive impairment by gut microbiota lipopolysaccharide production-suppressing *Lactobacillus plantarum* and *Bifidobacterium longum* in mice. Food Func.

[21] Kim H, Kim S, Park SJ, Park G, Shin H, Park MS (2021). Administration of *Bifidobacterium bifidum* BGN4 and *Bifidobacterium longum* BORI improves cognitive and memory function in the mouse model of Alzheimer's disease. Front Aging Neurosci.

[22] Lee M-C, Hsu Y-J, Chuang H-L, Hsieh P-L, Ho H-H, Chen W-L (2019). *In vivo* ergogenic properties of the *Bifidobacterium longum* OLP-01 isolated from a weightlifting gold medalist. Nutrients.

[23] Lin C-L, Hsu Y-J, Ho H-H, Chang Y-C, Kuo Y-W, Yeh T-S (2020). *Bifidobacterium longum subsp. longum* OLP-01 supplementation during endurance running training improves exercise performance in middle-and long-distance runners: a double-blind controlled trial. Nutrients.

[24] Sacco RL, Kasner SE, Broderick J, Caplan LR, Connors JJ, Culebras A (2013). An updated definition of stroke for the 21st century: a statement for healthcare professionals from the American Heart Association/American Stroke Association. Stroke.

[25] Greenberg SM, Ziai WC, Cordonnier C, Dowlatshahi D, Francis B, Goldstein JN (2022). 2022 guideline for the management of patients with spontaneous intracerebral hemorrhage: a guideline from the American Heart Association/American Stroke Association. Stroke.

[26] Kissela BM, Sauerbeck L, Woo D, Khoury J, Carrozzella J, Pancioli A (2002). Subarachnoid hemorrhage: a preventable disease with a heritable component. Stroke.

[27] Cuervo M, Ansorena D, Martínez-González MA, García A, Astiasarán I, Martínez JA (2009). Impact of global and subjective mini nutritional assessment (MNA) questions on the evaluation of the nutritional status: the role of gender and age. Arch Gerontol Geriatr.

[28] Ho DKN, Chiu W-C, Kao J-W, Tseng H-T, Lin C-Y, Huang P-H (2024). Reliability issues of mobile nutrition Apps for cardiovascular disease prevention: comparative study. JMIR Mhealth Uhealth.

[29] Chueh T-L, Wang Z-L, Ngu Y-J, Lin P-L, Owaga E, Hsieh R-H (2024). A mobile-based nutrition tracker App enhanced dietitian-guided 2:1:1 diet-induced weight loss: an 8-week retrospective cohort study in Taiwan. Nutrients.

[30] Wong A, Nyenhuis D, Black SE, Lorraine SN, Eugene SK, Pauline WL (2015). Montreal Cognitive Assessment 5-minute protocol is a brief, valid, reliable, and feasible cognitive screen for telephone administration. Stroke.

[31] Tombaugh TN (2004). Trail Making Test A and B: normative data stratified by age and education. Arch Clin Neuropsychol.

[32] Scarpina F, Tagini S (2017). The Stroop Color and Word Test. Front Psychol.

[33] Ng SS, Hui-Chan CW (2005). The timed up & go test: its reliability and association with lower-limb impairments and locomotor capacities in people with chronic stroke. Arch Phys Med Rehabil.

[34] Wist S, Clivaz J, Sattelmayer M (2016). Muscle strengthening for hemiparesis after stroke: a meta-analysis. Ann Phys Rehabil Med.

[35] Dunn A, Marsden D, Nugent E, Van Vliet P, Spratt NJ, Attia J (2015). Protocol variations and six-minute walk test performance in stroke survivors: a systematic review with meta-analysis. Stroke Res Treat.

[36] Callahan BJ, Sankaran K, Fukuyama JA, McMurdie PJ, Holmes SP (2016). Bioconductor workflow for microbiome data analysis: from raw reads to community analyses. F1000Res.

[37] Corrigan ML, Escuro AA, Celestin J, Kirby DF (2011). Nutrition in the stroke patient. Nutr Clin Pract.

[38] Talani G, Biggio F, Mostallino M, Locci V, Porcedda C, Boi L (2020). Treatment with gut bifidobacteria improves hippocampal plasticity and cognitive behavior in adult healthy rats. Neuropharmacology.

[39] Savignac H, Tramullas M, Kiely B, Dinan T, Cryan J (2015). *Bifidobacteria* modulate cognitive processes in an anxious mouse strain. Behav Brain Res.

[40] Bauer KC, Huus KE, Finlay BB (2016). Microbes and the mind: emerging hallmarks of the gut microbiota-brain axis. Cell Microbiol.

[41] Mu C, Yang Y, Zhu W (2016). Gut microbiota: the brain peacekeeper. Front Microbiol.

[42] Wang Q, Guo M, Liu Y, Xu M, Shi L, Li X (2022). *Bifidobacterium breve* and *Bifidobacterium longum* attenuate choline-induced plasma trimethylamine N-oxide production by modulating gut microbiota in mice. Nutrients.

[43] Lee J, d'Aigle J, Atadja L, Quaicoe V, Honarpisheh P, Ganesh BP (2020). Gut microbiota-derived short-chain fatty acids promote poststroke recovery in aged mice. Circ Res.

[44] Palm NW, De Zoete MR, Cullen TW, Barry NA, Stefanowski J, Hao L (2014). Immunoglobulin A coating identifies colitogenic bacteria in inflammatory bowel disease. Cell.

[45] Zhou C, Zhao H, Xiao X-Y, Chen B-D, Guo R-J, Wang Q (2020). Metagenomic profiling of the pro-inflammatory gut microbiota in ankylosing spondylitis. J Autoimmun.

[46] Ling Y, Gong T, Zhang J, Gu Q, Gao X, Weng X (2020). Gut microbiome signatures are biomarkers for cognitive impairment in patients with ischemic stroke. Front Aging Neurosci.

