# Supplementary material for: Effect of Bifidobacterium longum on cognition and microbiota in post-stroke patients: a single-blinded, controlled trial
Source: Int J Med Sci. 2026 Feb 11;23(3):1058–69. doi: 10.7150/ijms.124024 (PMC12965072; doi:10.7150/ijms.124024)
Supplement: Supplementary file 1 — Supplementary figures. [file ijmsv23p1058s1.pdf]

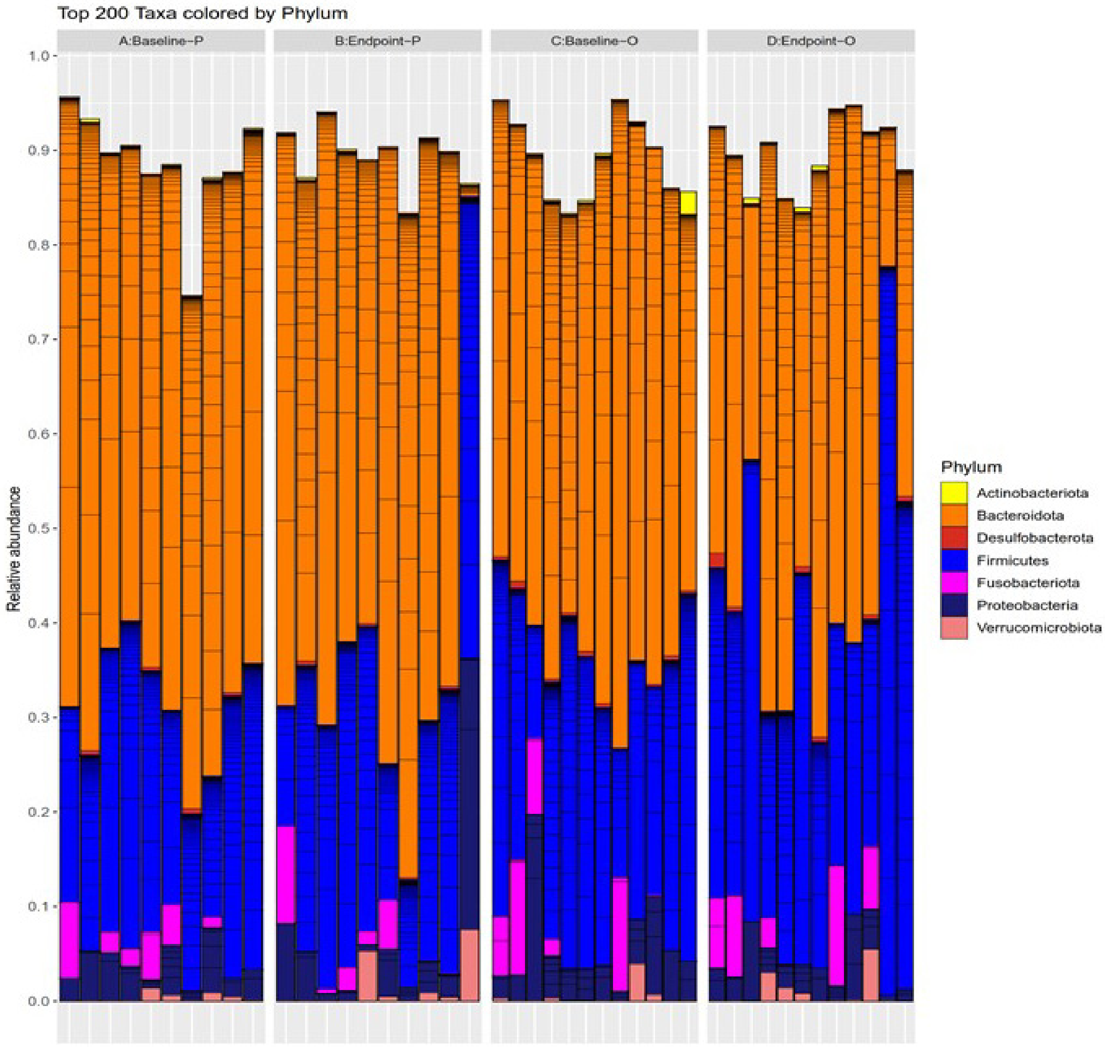

Supplementary Figure 1. Effects of *Bifidobacterium longum* OLP-01 on relative abundance of phylum in gut microbiota. A: baseline of the placebo group (n = 10), B: endpoint of the placebo group (n = 10), C: baseline of the OLP-01 group (n = 12), D: endpoint of the OLP-01 group (n = 12).

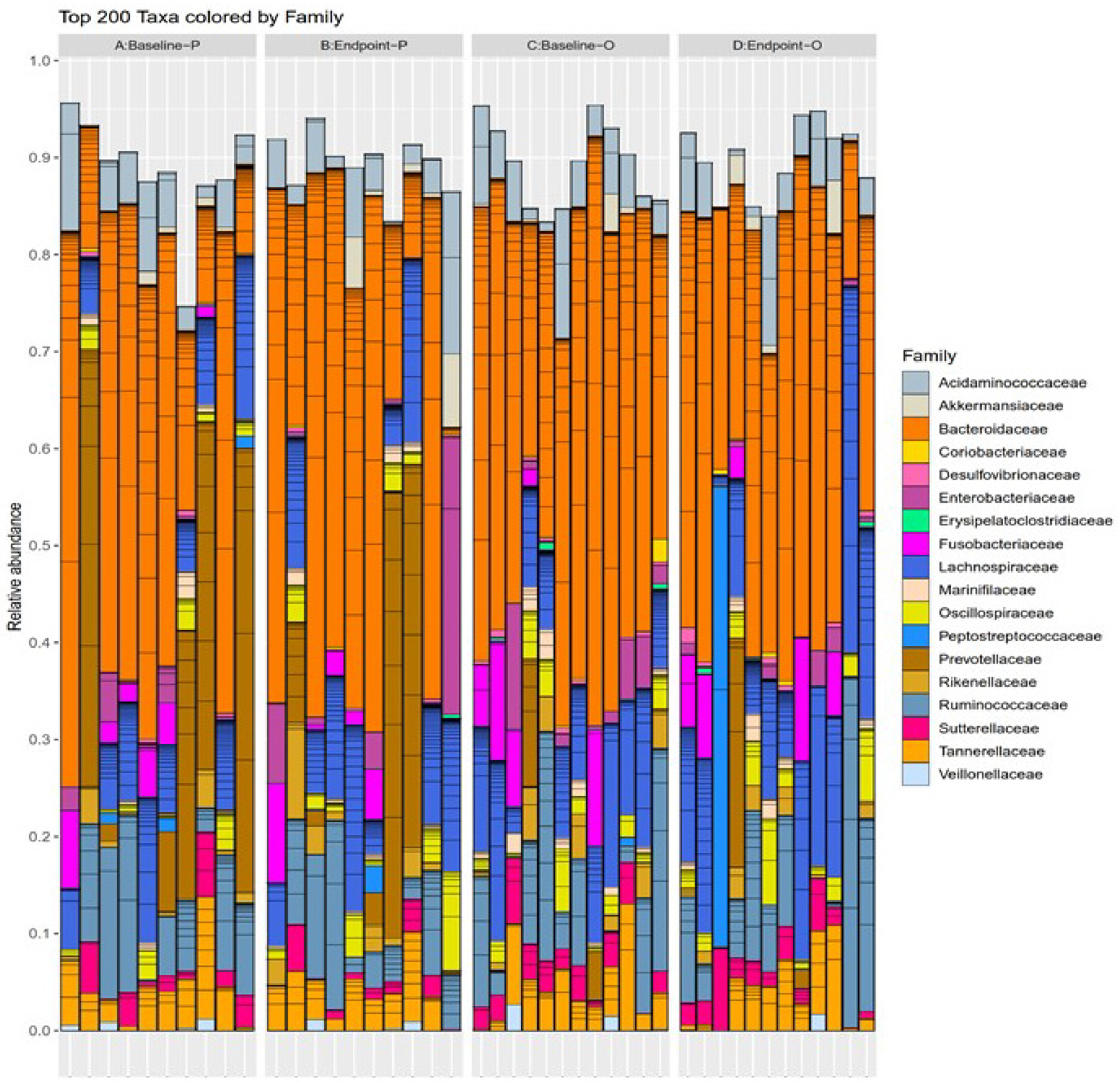

Supplementary Figure 2. Effects of *Bifidobacterium longum* OLP-01 on relative abundance of family in gut microbiota. A: baseline of the placebo group (n = 10), B: endpoint of the placebo group (n = 10), C: baseline of the OLP-01 group (n = 12), D: endpoint of the OLP-01 group (n = 12).

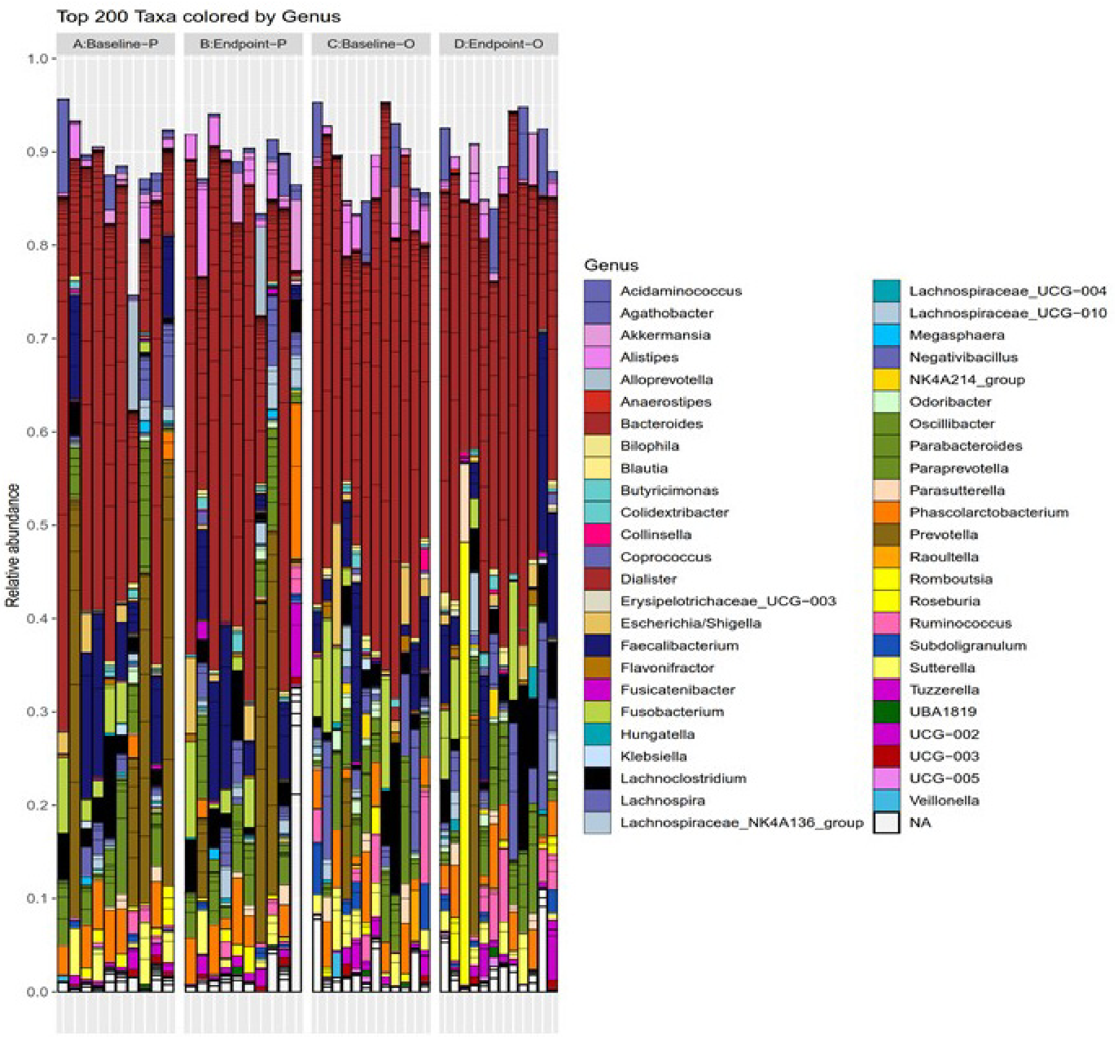

Supplementary Figure 3. Effects of *Bifidobacterium longum* OLP-01 on relative abundance of genus in gut microbiota. A: baseline of the placebo group (n = 10), B: endpoint of the placebo group (n = 10), C: baseline of the OLP-01 group (n = 12), D: endpoint of the OLP-01 group (n = 12).
